# Using developmental evaluation to enhance continuous reflection, learning and adaptation of an innovation platform in Australian Indigenous primary healthcare

**DOI:** 10.1186/s12961-020-00562-4

**Published:** 2020-05-12

**Authors:** Jodie Bailie, Alison Frances Laycock, David Peiris, Roxanne Gwendalyn Bainbridge, Veronica Matthews, Frances Clare Cunningham, Kathleen Parker Conte, Seye Abimbola, Megan Elizabeth Passey, Ross Stewart Bailie

**Affiliations:** 1 The University Centre for Rural Health, The University of Sydney, 61 Uralba Street, Lismore, NSW 2480 Australia; 2 The School of Public Health, The University of Sydney, Sydney, NSW 2006 Australia; 3 Menzies School of Health Research, Charles Darwin University, Casuarina, Australia; 4 The George Institute for Global Health, The University of New South Wales, Sydney, Australia; 5 Centre for Indigenous Health Equity Research, Central Queensland University, Rockhampton, Australia

**Keywords:** Developmental evaluation, innovation platforms, Indigenous health, continuous quality improvement, innovation, health systems strengthening, primary healthcare, multi-stakeholder networks, co-production, systems thinking

## Abstract

Effective efforts to strengthen health systems need diverse, multi-stakeholder networks working together on complex or ‘wicked’ problems such as prevention and control of chronic diseases, solutions to which go beyond the role and capability of one organisation. The contextual complexities inherent in ‘wicked’ problems mean that solutions warrant a systems approach that encompasses innovation and new ways of thinking about, facilitating and implementing collective decision-making processes and change practices.

Innovation platforms are a mechanism for facilitating communication and collaboration among diverse stakeholders, promoting joint action and stimulating innovation. Developmental evaluation is an approach that is increasingly being used to evaluate innovative and emergent programmes and projects, as it enables evaluators to provide real-time feedback so that evaluation findings can be used to guide development and adaptations. Developmental evaluation emphasises learning and adaptation, and aligns well with the implementation of innovation platforms that have continuous reflection, learning and adaptation as a specific design principle.

Here, we outline our rationale for applying a developmental evaluation to enhance the formation, functioning and outcomes of an innovation platform aimed at accelerating and strengthening large-scale quality improvement efforts in Australian Aboriginal and Torres Strait Islander primary healthcare. We provide examples to explain how the developmental evaluation findings were used for adaptation of the innovation platform and assess to what extent our application of developmental evaluation was consistent with, and reflective of, its essential principles.

Our evaluation aligned strongly with the principles of developmental evaluation, and the approach we took was well suited to situations with a developmental purpose, innovation niche and complexity such as innovation platforms. As a result, along with the increasing interest in multi-stakeholder platforms (e.g. innovation platforms) and the inherent challenges with evaluating these complex networks, we anticipate our use of this approach being of interest globally.

## Introduction

Effective efforts to strengthen health systems need diverse, multi-stakeholder networks working together on complex or ‘wicked’ problems such as prevention and control of chronic diseases, the solutions to which go beyond the role and capability of one organisation [1–3]. Promoted as a vehicle to stimulate and support multi-stakeholder collaboration, ‘innovation platforms’ are considered particularly useful when there are complex, system-wide issues requiring coordinated action and collective problem solving [4, 5].

As their name indicates, the objective of innovation platforms is innovation, which is stimulated when people come together to learn, share ideas and solve problems. Features that distinguish innovation platforms from other types of networks include bringing together people from different parts of the system to ensure a diverse stakeholder composition, and having shared goals and interests along the supply chain to focus on problem solving within complex systems [4]. Innovation platforms have been widely adopted in the agricultural research and development sector, mainly in Africa, but have only recently been applied to the health sector [4–6]. Given the novelty of this concept in health, rigorous and critical evaluation is required [4].

Developmental evaluation (DE) is increasingly being used as an approach to evaluate innovative and emergent programmes and projects [7, 8]. This is because it allows evaluators to provide rapid feedback to programme implementers who can then use the evaluation findings to guide programme changes and adaptations. Such an approach aligns well with the implementation of innovation platforms that have continuous reflection, learning and adaptation as specific design characteristics. In addition, there is an acknowledged gap in the literature on appropriate monitoring, evaluation and learning approaches to support innovation platforms [5, 9, 10]. There is also limited information on how collaborative and co-productive health research can be done effectively, including a lack of evaluation of collaborative research models more broadly [11].

In this paper, we outline the rationale for applying DE to enhance the formation, functioning and outcomes of an innovation platform in Aboriginal and Torres Strait Islander primary healthcare (PHC). We do this by providing an overview of the innovation platform, explaining the fundamentals of a DE, and describing the methods we used in implementing the DE by assessing our approach against its essential principles. Given the focus on ‘learning and adaptation’ in taking such an approach, and the subsequent emergent design of the DE, it was neither possible nor appropriate to detail a priori the specific methods used. Thus, in this paper, we describe the rationale for our approach and how it aligned with DE methods and innovation platform functions. We also provide examples to explain how the DE findings were used to adapt the innovation platform’s functioning.

### An innovation platform: Centre for Research Excellence in Integrated Quality Improvement

As with other colonised populations worldwide, Aboriginal and Torres Strait Islander Australians (hereafter respectfully referred to as Indigenous, acknowledging cultural and historical diversity) experience worse health outcomes and shorter life expectancy than non-Indigenous Australians. These inequities are a pervasive legacy of colonisation, land dispossession, displacement, disempowerment, social and economic exclusion, and ongoing racial discrimination [12]. Understanding and addressing the complexity of the causal relationships that underlie the health conditions of Indigenous Australians requires an innovative systems approach to thinking about, facilitating and implementing collective decision-making processes and change practices.

Recognising the importance of quality improvement initiatives in Indigenous PHC, the National Health and Medical Research Council of Australia funded a Centre for Research Excellence in Integrated Quality Improvement in Indigenous PHC (CRE-IQI) [4] for 5 years from 2015 to 2019. The stated vision of the CRE-IQI was to improve Indigenous health outcomes by accelerating and strengthening system-wide PHC through supporting quality improvement efforts at health service, regional and national levels.

To support this vision, and to be consistent with the operationalisation of an innovation platform, the CRE-IQI had, from its inception, embraced a range of organisations and people working in diverse roles and at different levels of the health system. They included researchers from universities and research organisations, policy officers from State and Territory health departments, managers and practitioners from State/Territory-level support organisations established for Indigenous PHC services, and health practitioners from both Indigenous community-controlled and government-managed health services. Based on the literature [5, 13, 14] and our own experience [4], Table 1 outlines the key functions of an innovation platform [4, 5, 13], and describes how the CRE-IQI innovation platform’s activities and aspirations fulfilled these functions.

**Table 1:** Key functions of the CRE-IQI as an innovation platform

Key functions of an innovation platform	CRE-IQI innovation platform aspirations and activities undertaken to fulfil key functions
Linking people from all levels of a system	Brought together people working at all levels of the health system with researchers, policy-makers and practitioners from Indigenous PHC services
Identifying shared goals and interests, common problems and solutions	Collaborated to develop the vision, research aims, priority projects for resource allocation and cross-cutting programmes of the CRE-IQI network
Leveraging research and/or expertise	Utilised members’ knowledge to leverage new resources, implement collective and coordinated action, and advocate for policy change
Enabling long-term learning and capacity-strengthening	Developed health research workforce capacity by sharing problems and experiences, developing learning opportunities and networking – adopting an ‘all teach, all learn’ approach [15]
Establishing effective governance	Set up a project coordinating centre (the CRE-IQI) and management committee to support and drive these key activities and provide high-level strategic direction and oversight
Encouraging continuous reflection, learning and adaptation	Implemented a developmental evaluation to support continuous reflection, learning and adaptation
Out-scaling and up-scaling knowledge to broaden impact	Facilitated horizontal diffusion of innovations by broadening the application (or ‘out-scaling’) of quality improvement to non-clinical areas of PHC through implementing, testing and improving its application; facilitated up-scaling innovations by embedding them at higher levels of the health system and other sectors
Generating and sharing knowledge	Established the innovation platform itself to be a vehicle for integrated research and knowledge translation, with research, translation and learning occurring in the exchanges and interactions of service providers, policy-makers and researchers

*CRE-IQI* Centre for Research Excellence in Integrated Quality Improvement, *PHC* primary healthcare

The CRE-IQI held biannual face-to-face meetings to provide its members with opportunities to progress project development and research translation, hear about project outcomes, share ideas and build relationships. It established cross-cutting programmes to strengthen research capacity, collaboration and research translation. Webinars and teleconferences enabled members located across Australia to connect and engage with leaders in PHC and Indigenous research, and masterclasses were offered around each biannual meeting to increase members’ skills and knowledge. A detailed outline of the aims and cross-cutting work programmes of the CRE-IQI, and how it functioned as an innovation platform, is available in other papers [4, 16].

**Fig. 1:**
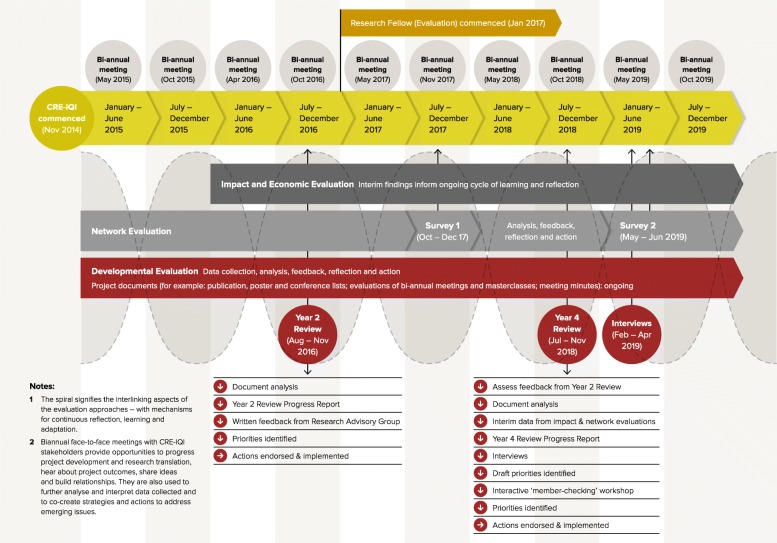
Timeline of developmental evaluation activities, demonstrating linkages between evaluative approaches. *CRE-IQI* Centre for Research Excellence in Integrated Quality Improvement

Because of the inherent challenges with evaluating complex networks (including innovation platforms), we designed a mixed-methods, multi-pronged evaluation, with three complementary and partly overlapping components [4] – the DE, a network evaluation, and an impact and economic evaluation [17]. The DE drew on early findings from the other two components to shape the functioning of the innovation platform. Details of the methods and findings from the network evaluation, and impact and economic evaluation will be reported separately. In Fig. 1, we show the interlinking aspects of the evaluation approaches, with a specific focus on the methods of data collection for the DE. This figure is further discussed in relevant sections of this paper.

DE was identified both as a way of attending to the complexity of evaluating the innovation platform, and as a way of supporting its design tenet of continuous reflection, learning and adaptation. Drawing on a range of data (e.g. administrative records, stakeholder interviews, network analysis surveys, impact metrics) to synthesise and apply lessons from the formation, functioning and outcomes of the innovation platform, the DE approach informed the innovation platform’s operations, work programmes and future directions.

### Developmental evaluation: supporting innovation and adaptation

DE was first described by Patton in the mid-1990s as a distinct approach to evaluation with the explicit purpose of helping to develop and shape an innovation, intervention or programme that is emergent, complex and dynamic [18]. Unlike traditional forms of evaluation, the focus of DE is on reflection, learning and change to enable interventions to adapt to the emerging complex environments in which they are situated [7, 18]. There are clear distinctions between DE and formative evaluation. Formative evaluation focuses on informing the planning of a defined initiative, tends to be conducted prior to or at an early stage of the initiative, and is aimed at improving, enhancing and standardising the initiative. By contrast, DE is applied throughout the life of a developing or emerging initiative in which knowledge is uncertain and/or the evidence base is under-developed or not clearly relevant. A DE enables the work to adapt quickly to any changes in the environment or to new learnings that emerge, thereby also generating and advancing knowledge about the intervention in the field.

DE is an approach rather than a specific method. Methods used within a DE approach intend to be evolving and flexible, with dynamic designs as the intervention unfolds. Unlike conventional evaluations that require fidelity to particular models or methods, a DE draws on evaluation knowledge, core ideas of innovation, complexity concepts and systems thinking to develop and apply the evaluation in context. The evaluation thus becomes part of the intervention as data are systematically gathered, interpreted and reported in a timely way to ensure that results are useful for end-users (or innovators). In 2016, Patton further developed his research in this area by identifying eight inter-related and mutually reinforcing principles of DE to guide this way of working [7]. These principles are described in Table 2.

**Table 2:** The eight principles of developmental evaluation

DE principles	Brief description of DE principles
Developmental purpose	The focus is on informing and supporting an innovation in its development, thereby differentiating DE from traditional evaluation methods that seek to assess the degree to which goals/aims were achieved
Innovation niche	DE is only possible if innovation is present or if efforts are being made to institute it
Complexity perspective	Adaptive evaluation design and processes enable the identification and analyses of emergent findings
Systems thinking	Key to DE is employing systems thinking to frame, design and address complex problems while attending to boundaries, perspectives and interrelationships
Evaluation rigour	To be credible and useful, DE needs to employ both rigorous thinking and evaluation methods
Co-creation	Simultaneously developing the innovation and the evaluation with diverse stakeholders stimulates and streamlines the change process
Utilisation focus	A strong utilitarian focus ensures that findings are useful for end-users
Timely feedback	Iterative, progressive processes ensure that feedback is ongoing and prompt to maximise utility

*DE* developmental evaluation. Table based on Patton et al. [7] and Patton [8].

### Systematically applying developmental evaluation within the innovation platform

Drawing on team reflections and discussions, and by providing examples, we describe how our application of DE to an innovation platform reflected the principles of a DE approach (as listed in Table 2).

#### Developmental purpose – an ‘improving’ rather than a ‘proving’ approach

The CRE-IQI evaluation had a developmental purpose in that it aimed to inform and support the formation, functioning and outcomes of the innovation platform. Dedicated resources were allocated to gathering data that would provide feedback and support developmental decision-making and adaptations along the emergent path. This evaluative approach aligned with the need for innovation platforms to have a mechanism for continuous reflection, learning and adaptation. Examples of decisions and adaptations made in response to evaluation feedback are listed in Table 3.

**Table 3:** Examples of evaluation feedback, team decisions and adaptations

Evaluation findings	Decisions and adaptations
Increase the number, input and attendance of health service providers input and attendance at CRE-IQI bi-annual meetings	CRE-IQI bi-annual meeting agendas were amended to include ‘health service showcases’, in which health services staff could present their quality improvement work and discuss opportunities for research collaboration and knowledge translation. Presentation topics were determined through consultation processes at previous bi-annual meetings and with the management committee. The CRE-IQI funded health services staff members to attend these meetings, which were held in different locations to encourage participation by a range of groups
Increase attendance by Indigenous stakeholders at bi-annual meetings	Personalised invitations to bi-annual meetings were extended to Indigenous stakeholders via telephone rather than emails, with funds specifically allocated for Indigenous stakeholders to attend. Increased steps were taken to ensure a culturally safe environment at the meetings, including their formal opening and closing by Indigenous people
Explicitly promote the shared values and working principles of the CRE-IQI	The principles of practice of the innovation platform were highlighted and discussed at the start of all bi-annual meetings, and purposely applied when developing criteria for the allocation of funds for CRE-IQI activities, such as ‘seed grants’ to develop research
Focus on capacity-strengthening, particularly in relation to Indigenous direction of, and participation in, research	Dedicated funding was allocated for a 12-month ‘research capacity-building’ position and a lead group to oversee and provide guidance on capacity-building strategies and activities. The terminology was changed to ‘capacity-strengthening’ in recognition of the existing strengths and knowledge among stakeholders, and monthly online research capacity-strengthening meetings held using ‘Zoom’ software
Grow Indigenous leadership in CRE-IQI management and research	An additional Indigenous researcher was appointed to the CRE-IQI leadership. Purposeful encouragement of co-leadership arrangements was established, whereby all new research grants and projects were to have an Indigenous leader or a co-leadership arrangement with a non-Indigenous member of the team
Enable Indigenous members to engage in CRE-IQI direction and governance despite their high workloads and multiple leadership commitments	The decision was made to embed and disperse Indigenous leadership and participation across all levels of the innovation platform, rather than having one overall reference group. This included the appointment of an Indigenous researcher to the leadership team; purposeful engagement and funding to increase attendance by Indigenous people and organisations at all meetings; greater focus and attention on embedding the ‘principles of practice’ established at the start of the innovation platform; and co-leadership arrangements with Indigenous and non-Indigenous collaborators on all projects
Provide information to CRE-IQI stakeholders through mechanisms such as meetings, new publications and news from the network	Based on feedback, a monthly email to all CRE-IQI stakeholders was established that would later become a regular and official online CRE-IQI newsletter
Boost engagement with, and readership of, the CRE-IQI newsletter	To improve readability and engagement, the monthly newsletter was adjusted to include more illustrative material and articles from stakeholders. Following research into the most effective dissemination times, the monthly newsletter was disseminated on a Tuesday or Thursday at either 10 am or 2 pm
Ensure that administrative data collected by the CRE-IQI (e.g. attendance at bi-annual meetings, publications, grants awarded) is capable of the required data analysis	Data collection procedures were reviewed, specifically, what was being collected and how, and, importantly, what aspects would need to be reported and the aggregations required. Changes were made and standard nomenclature adopted
Increase the focus on and engagement in research translation	Research translation strategies were prioritised and developed over a series of workshops at bi-annual meetings and discussions at management committee meetings. CRE-IQI stakeholders were provided with training opportunities in knowledge translation skills, including the use of social media, influencing policy and other relevant topics. CRE-IQI social media accounts were established and reviewed, resulting in increased use of Twitter to communicate research activities and findings. A dedicated research translation working group was convened and a position established to support projects and translation across the CRE-IQI. In the final year, knowledge synthesis workshops were held in which members collaboratively identified and prioritised the overall findings and key messages from CRE-IQI research. Research translation products were produced in a range of formats targeting different audiences
Respond to CRE-IQI stakeholders’ identified need for training in a range of relevant topics	Training needs were addressed through the establishment of webinar research seminars and face-to-face masterclasses. At each bi-annual meeting, participants were invited to suggest further topics to meet their professional development needs, such as social media training to extend research translation, engaging policy-makers in dissemination of research findings and using Indigenous methodologies
Strengthen CRE-IQI engagement with policy-making processes	Resources were directed into writing targeted policy and parliamentary submissions that drew on CRE-IQI research. Policy masterclasses were offered to members early in the CRE-IQI’s establishment and again in its final year. Relevant policy-makers were invited to bi-annual meetings with the aim of having their input into the development of research products, such as key messages, and of building relationships with them over an extended period. Final products of research projects included policy briefs, and the publication of a summary of overall policy messages from the CRE-IQI’s research. At the end of its funding period, the CRE-IQI targeted key policy-makers for briefings about the research findings
Prioritise further collaborative research in Indigenous primary healthcare quality improvement	Collaborative processes were undertaken to identify and refine the research priorities. These processes included presenting and working up ideas at bi-annual meetings, discussing research needs in management committee meetings and holding a series of smaller more focused workshops. A decision was made to develop a submission for funding beyond the innovation platform, with revised leadership arrangements to reflect the DE outcomes. This resulted in a proposal for a collaborative research network led by an Indigenous chief investigator, with 50% of the leadership team identifying as Indigenous

*CRE-IQI* Centre for Research Excellence in Integrated Quality Improvement, *DE* developmental evaluation

Many innovation platform stakeholders had a history of working in quality improvement and participatory action research, and this provided a foundation for understanding some of the key concepts and processes used in DE. We collected and interpreted data, worked out change strategies, implemented them, evaluated how they worked and repeated the cycle with different sets of data and feedback. To do this, we used iterative cycles of development and testing that could be compared with the ‘Plan – Do – Study – Act’ method common in continuous quality improvement. The congruency between quality improvement and DE has been identified by Laycock et al. [19].

#### Innovation niche

An issue explored by the CRE-IQI innovation platform members was to define what innovation meant to them. For the purposes of the CRE-IQI, it was agreed through collaborative processes that the most appropriate definition of innovation was one that emphasised the “*non-directed, organic sharing of ideas and practices*’ ( [2], p. 207). Innovation, therefore, was composed of information, that is, learning through the exchange of ideas and the production of knowledge. However, continuous innovation and adaptation were required in how “*learning through the exchange of ideas*” was achieved as the collaboration evolved. However, with no examples in the literature to guide the implementation of an innovation platform in health, we also had to be innovative in our use of the innovation platform concept.

#### Complexity and systems thinking perspectives – attending to non-linearity, feedback, emergence, relationships, boundaries and adaptation

The complexities of Indigenous PHC environments in relation to continuous quality improvement, and the multiple stakeholders engaged with the innovation platform, meant that an emergent evaluation design that did not predefine the innovation platform was required. We needed scope to move away from a ‘what is planned needs to be achieved’ mindset to one that could continually adapt based on what we were learning. Given this non-linearity, the DE focused on the development of the innovation platform in an evolving context. We used opportunistic and planned iterative cycles of reflection and analysis to understand how, and how well, the innovation platform was functioning, and how it could be adapted in rapid-time to function more effectively. Our bi-annual meetings with stakeholders were a vehicle for the DE to identify emerging issues through consultation and discussions, and also to present back, discuss and refine proposed modifications based on stakeholder feedback.

Given the complex environment of the innovation platform, a systems thinking approach assisted us in gaining deeper insights into how best to adapt its formation and functioning. Engaging multiple perspectives, whilst paying attention to relationships and interactions, was a key design feature of the DE approach. We examined how participants in the innovation platform learn from and influence each other, and paid attention to those activities/events that are catalysts for relationship development (i.e. biannual meetings, funding for new grant development and other mechanisms that encourage ‘dynamics’ to develop).

Conclusions were rendered through a collaborative and interactive process involving stakeholders, leading us to modify CRE-IQI strategies and processes. These collaborative change decisions were recorded in evaluation logs and detailed in project records such as minutes of meetings and agendas. Given that the innovation platform was an ‘open collaboration’, we were sensitive to examining how the scope of the research and membership expanded or changed over time – beyond PHC contexts to policy and the social determinants of health; therefore, we examined the characteristics of participation in the innovation platform to understand any changes in boundaries and representation.

#### Evaluation rigour

An evaluation working group was established to guide the comprehensive evaluation of the innovation platform, including the DE. The group was comprised of those researchers implementing evaluations within the innovation platform – specifically, network evaluation, impact and economic evaluation, and DE – and other stakeholders with specific expertise in evaluation. Initially, the evaluation working group was virtual. However, as the work progressed, there was agreement that more regular focused meetings were needed to bring together the evaluation streams, streamline the data collection, implement a group analysis of emerging data, and provide evaluation project management oversight. From mid-2017, fortnightly teleconferences were facilitated by the developmental practitioner and 6-monthly face-to-face meetings were held. The methods of data collection for the DE included document review, the Year 2 and Year 4 Reviews, and key stakeholder interviews as detailed below, while an overview of the timelines, data collection sources and methods, and feedback processes can be found in Fig. 1.

Given the range of functions required of the developmental evaluator, strong methodological skills were required as well as experience with a wide range of methods. Having the DE embedded within a broader evaluation working group enhanced the methodological rigour and provided exposure to different evaluation methods. Because the DE was situated within a broader evaluative strategy (Fig. 1), which included an impact evaluation, this helped to alleviate concerns that we had not developed a programme logic/theory of change at the start of the innovation platform. Instead, we worked to generate evidence in real time through flexible, situationally tailored evaluation design.

Members of the evaluation working group had some experience with applying DE techniques [19]. However, because of the uncertainty inherent in DE, and the paucity of literature describing the methods used within it, as a team, we had to reflect regularly on whether our evaluation was indeed developmental. The evaluation working group offered a forum for this reflection to occur.

##### Document review

Administrative project records were used to provide ongoing intelligence on the CRE-IQI innovation platform development and context. Data sources included minutes from the management committee, bi-annual stakeholder meetings, publication, poster and conference lists, attendance lists and evaluations of bi-annual meetings, masterclasses and research capacity-strengthening teleconferences, research project applications, and student projects. Reports of other evaluation activities, such as the network evaluation, also provided data for the DE. These documents were then used to identify and clarify key issues, dates, events and tasks, and to track major decisions and developments in the innovation platform formation, functioning and outcomes.

##### Year 2 and Year 4 Reviews

Major activities of the DE were the Year 2 and Year 4 Reviews of the CRE-IQI, with the latter building on the learning and feedback from the former. The goal of both reviews was to obtain input on the progress to date of the CRE-IQI in terms of outputs and achievements; the key messages emerging from the CRE-IQI’s collaborative research; assessing the extent to which the CRE-IQI was meeting its aims; and how best to optimise the ongoing operation of the CRE-IQI.

The Year 4 Review had the additional aim of reviewing progress on addressing key issues identified in the Year 2 Review and identifying priorities for the remaining 15 months of the CRE-IQI. Another major focus was the way in which Indigenous leadership and participation were being enacted and identifying steps that could be taken to strengthen this aspect of the CRE-IQI’s work.

The scope of the Year 4 Review was collaboratively determined with the CRE-IQI management committee and included an analysis of feedback about the Year 2 Review process and report presentation. While both reviews developed reports to aid consultation and change processes, the Year 4 Review employed more active processes to gain feedback; these included interviewing key members of the innovation platform (*n* = 28) along with several external stakeholders (*n* = 36) (see Additional file 1 for interview questions). In addition to informing the final stages of work for the CRE-IQI, the Year 4 Review was intended to inform ongoing collaborative projects extending beyond its current lifespan. Figure 1 has a description of the methods used for the Year 2 and Year 4 Reviews.

The process of undertaking the Year 2 review identified several data management systems that needed to be established, or refined, to ensure that administrative data were collected in a timely and accurate manner. It was found, for example, that the way in which data were being entered into Excel spreadsheets made analysis for the Year 2 review more difficult, so the data entry system was adjusted accordingly.

##### Interviews

As noted, interviews (*n* = 28) were conducted as part of the Year 4 Review, with a further round of interviews (*n* = 36) undertaken to explore emergent themes from the review (Additional file 2). Participants who were purposively sampled to obtain a broad range of perspectives from different organisations included CRE-IQI researchers, members of the management committee, and several national and international participants from bi-annual meetings, teleconferences and projects.

##### Ethics

Obtaining ethics approval to undertake the DE allowed for the evaluation questions to be developed in response to emerging priorities and for appropriate methodologies to be implemented. The University of Sydney Human Research Ethics Committee (Project 2018/206) and the Human Research Ethics Committee of the Northern Territory Department of Health and Menzies School of Health Research (Project 2018–3105) approved the DE.

#### Co-creation

The CRE-IQI innovation platform concept and the DE were developed and refined together drawing on input from multiple stakeholders and on purposeful opportunities to garner further Indigenous input. Placing importance on context, and valuing Indigenous knowledge by centring the voices of participant populations in the research, data analysis processes occurred collaboratively to capture a variety of worldviews that also embedded ‘member checking’ processes (see Section above on compexity and systems thinking perspectives).

This collaborative data analysis approach provided immediate, useable feedback to engage CRE-IQI stakeholders in co-creating solutions, thus reflecting some of the strong principles of DE. The learnings and actions from the innovation platform were guided by facilitated reflection and analysis processes that drew on data collected as part of the DE as well as stakeholders’ experiences and feedback. Questions used to guide these processes were: what? (what happened?), so what? (what do the results mean or imply? how did we influence the results?), and now what? (how do we respond? what should we do differently?). We focused on documenting the change decisions and on using collaborative analysis processes with CRE-IQI members to analyse and interpret the collected data further and to co-create strategies and actions to address emerging issues.

##### Embedded, not detached – the active role of the evaluator

Consistent with a DE approach, a research fellow (evaluation), aka the DE practitioner (JB), was embedded within the innovation platform team. This meant that any changes to its direction and evaluation – based on insights, learnings and critically reflective conversations between the evaluator and CRE-IQI management and members – could be facilitated rapidly as needs emerged.

The CRE-IQI innovation platform was operationalised through a project coordinating centre, which meant there was dedicated resourcing for part-time positions in both project management and project administration. During the ‘set-up’ phase of the innovation platform, the DE had been envisaged as being the responsibility of the innovation platform’s project manager, as there was a significant amount of work needed to establish agreements, policies, procedures and governance structures. However, based on evaluative data, in late 2016, it was agreed by the management committee to reshape the project manager’s role so that its primary focus was on implementing the DE and project management was secondary to the role. In January 2017, a research fellow (evaluation) (JB), aka the DE practitioner, was embedded within the team to lead the DE and to coordinate it with the other evaluation activities (Fig. 1), along with project management responsibilities. Restructuring the project manager position to predominately focus on the DE, with support from a project administrator, enabled us to handle large volumes of data. Although this investment in resourcing was reasonably small compared to the overall project budget, it did give us dedicated personnel who were both embedded within the team and able to action identified adaptations based on ongoing data collection and analysis processes. Being embedded with a dual role of DE practitioner and project management allowed the evaluator to be present at management committee meetings as well as at the evaluation working group and other meetings. Having the DE practitioner as a core member of the team enabled everyone on it to build a deep understanding of issues and to act in a timely manner.

Defining the boundaries between the DE practitioners role and project management was difficult and boundaries were often blurred. Having the DE practitioner role embedded within a broader evaluation working group (as detailed above), enabled the DE practitioner to liaise with a network of peers to progress the role and also to access mentoring from an evaluation working group member who was an experienced DE practitioner.

#### Utilisation focus – ensuring findings are useful for end-users

The innovation platform was a vehicle for integrated research, knowledge generation and sharing. Research, translation and learnings were intended to occur during structured and informal interactions between health service providers, policy-makers and researchers. Ensuring that our evaluation findings were useful was paramount, not least because many of our end-users were participants in the innovation platform.

Because the process focused on utilisation, making sense of emergent findings involved the evaluator working with innovation platform participants to analyse and understand the data, for example, the presentation of emergent findings from the Year 4 Review to the management committee, evaluation working group and to the broader network at the bi-annual meetings. The findings were further synthesised and prioritised during these interactions, and strategies to address them were identified through collaborative processes, for example, by revising the research project guidelines to increase Indigenous leadership of research.

#### Timely feedback

Feedback occurred at pre-determined times, such as part of the planned Year 2 and Year 4 reviews, as well as opportunistically when it emerged that change decisions were required (for example, through discussions at bi-annual or management committee meetings). Timely feedback to CRE-IQI management and governance was essential to ensure that evaluation data could be used to strengthen the formation and effective functioning of the innovation platform.

#### Learning through knowledge exchange

In addition to reflecting the eight DE principles described by Patton, we observed that evaluating the innovation platform developmentally allowed for the acquisition of new knowledge and skills through multiple interactions with stakeholders. This ‘learning through knowledge exchange’ aligned well with one of the key elements of innovation platforms, which is “*to enable long-term learning and capacity strengthening*” and “*knowledge generation and sharing*” [4]. This design element went beyond co-creation because it emphasised the ongoing development of a learning culture.

## Conclusion

The purpose of this paper is to provide a practical example of a DE by outlining the methods of applying it to an innovation platform in an Indigenous PHC setting. Although DE is gaining some traction and becoming recognised as a distinct and useful approach, it is also relatively new, so theory and practice are evolving [20]. As outlined above, in an attempt to define when an evaluation can be called developmental, Patton developed eight defining principles that should be evident [7, 8]. In assessing our approach against these eight principles, we found strong concordance between our DE and the principles he identified. However, because of the interrelatedness of these DE principles, it proved challenging to demonstrate adherence to each of the principles without being duplicative in explanation.

There is a nascent recognition of the suitability of DE in Indigenous contexts [19, 21–23], as it attends to complexity and systems thinking. Our experience with DE also shows it to be a good fit for innovation platforms that need to have continuous reflection and learning. Furthermore, DE embraces situations that have a developmental purpose, innovation niche and complexity such as innovation platforms.

To our knowledge, DE has not previously been applied to innovation platforms. We acknowledge, however, that previous evaluations might have had some of the features of a DE, but that these have either not been labelled as such or have not been the focus of a publication. The insights provided here will be developed further when the DE findings are outlined and discussed in future publications. Even though this example is focused on an innovation platform in the Indigenous Australian PHC context, we expect it will be useful in other contexts because of the increasing interest in multi-stakeholder platforms (such as innovation platforms) and the inherent challenges with evaluating these complex networks.

## Supplementary information

**Additional file 1.** Interview guide for Year 4 Review.

**Additional file 2.** Interview guide for further interviews exploring emergent issues related to the innovation platform.

## Data Availability

Not applicable.

## Abbreviations

CRE-IQI: Centre for Research Excellence in Integrated Quality Improvement
DE: developmental evaluation
PHC: primary healthcare
