# Supplementary material for: Using developmental evaluation to enhance continuous reflection, learning and adaptation of an innovation platform in Australian Indigenous primary healthcare
Source: Health Res Policy Syst. 2020 May 12;18:45. doi: 10.1186/s12961-020-00562-4 (PMC7218558; doi:10.1186/s12961-020-00562-4)
Supplement: Supplementary file 1 — Additional file 1. Interview guide for Year 4 Review. [file 12961_2020_562_MOESM1_ESM.docx]

Additional file 1: Interview guide for Year 4 Review

1. How and why did you become involved in the CRE-IQI?
2. *Enacting the key principle of Indigenous leadership and participation:* We welcome practical advice on specific steps or activities that we can take to strengthen this aspect of our work.
3. *Key messages:* We welcome comments on the significance, appropriate framing and relevance of the key messages emerging from the CRE-IQI’s work.
4. *Emerging areas of research:* We welcome comments on: a) What you see as the priorities in terms of further CQI-related research, particularly in relation to health service and systems development in Australia and internationally; b) Given your knowledge of the CRE-IQI, which priorities are we best able to address and why?
5. *Innovations*: Do you have any advice or suggestions on a) How we have tailored the definition of ‘innovations’ for the purposes of the CRE-IQI; b) How we have categorised innovations (innovating ideas and processes and innovating for scaling-out). From your reading of the review are there any other innovations that have emerged from the CRE-IQI and how would they be best communicated?
6. Do you have any comments on outputs and achievements to date in relation to the cross-cutting themes of research translation, research capacity strengthening and collaboration?
