# Supplementary material for: Using developmental evaluation to enhance continuous reflection, learning and adaptation of an innovation platform in Australian Indigenous primary healthcare
Source: Health Res Policy Syst. 2020 May 12;18:45. doi: 10.1186/s12961-020-00562-4 (PMC7218558; doi:10.1186/s12961-020-00562-4)
Supplement: Supplementary file 2 — Additional file 2. Interview guide for further interviews exploring emergent issues related to the innovation platform. [file 12961_2020_562_MOESM2_ESM.docx]

Additional file 2: Interview guide for further interviews exploring emergent issues related to innovation platform

| **Elements** | **Theme** | **Issues to reflect upon** | **Interview prompts** |
| --- | --- | --- | --- |
| Innovation platform:  - Establishment[1]  - Structure[2] | Composition and initiation of platform[3] | Representation and composition[3] | How did the CRE-IQI come about? How did you become involved and when?  Were you involved in the previous ABCD Partnership or other existing networks?  Prompt: Building on existing networks or are new networks created?  Who participates and how?  Can you comment on the diversity among CRE-IQI members^1^ (i.e. different backgrounds/organisations)  How are Indigenous people and organisations represented in the CRE-IQI?  What works well and why in terms of the CRE-IQI’s composition? |
|  |  | Common objective[3] | Level of awareness and understanding of the critical issue being addressed by the CRE-IQI  Objective of the CRE-IQI and how it was defined with stakeholders?  How have different needs and interests been taken into account?  Does the innovation platform objective now differ from the (initial) project vision? |
|  |  | Relevant research questions[3] | Are stakeholders sufficiently empowered to articulate their demands? How and by whom are research questions identified?  Are stakeholders’ needs effectively translated into a coherent set of relevant questions?  How, where and by whom is research conducted?  When and how research findings are made available? [Note: CRE-IQI research translation]  Have Indigenous research priorities led the agenda setting, and how? |
|  |  | Development of principles[4] | Are you aware of the development of a set of principles to guide how the CRE-IQI operates?  How did this come about? How were the principles identified? |
| Innovation platform:  - Functioning[1]  - Conduct[2] | Coordination and facilitation of the platform[3] | Principles guiding functioning[4] | What have these principles meant to you?  Are the principles meaningful? Why have these principles been important to the CRE-IQI?  What does implementation of the principles look like in practice?  Have there been critical points in the lifecycle of the CRE-IQI where the principles have been modified in response to emerging issues/context? If so, please outline.  (Prompts: co-leadership arrangements; the “All teach, all learn” approach; authorship; guiding research; participation in bi-annual meetings; Indigenous advisory group, etc.) |
|  |  | Facilitation[3] | How do you see the CRE-IQI being facilitated?  What works well from your perspective and why? What doesn’t work well and why? Examples.  [Probe: Indigenous facilitation]  Perceptions of communication channels within the CRE-IQI? |
|  |  | Knowledge co-creation[3] | Perceptions of the CRE-IQI’s “All teach, all learn” approach (valuing both Indigenous and non-Indigenous knowledge)  Does the CRE-IQI support and build on “collective learning”? ^1^ Examples.  Is knowledge co-created? If so how and if not, why? Examples.  How are reflection and discussion stimulated? ^1^ |
|  | Culture of the CRE-IQI[3, 5] | Power asymmetries[3] | Are existing power structures within the innovation platform explicitly identified, addressed and attended to? How and why? What are they?  How does decision-making work? Is it top down or bottom up (or across)?  Is sufficient time and attention given to building relationships? How does this occur and what enables or constrains this? |
|  |  | Conflicts negotiations and trust [3] | Are visible as well as invisible conflicts within the CRE-IQI recognised and effectively dealt with?  Is sufficient time and attention paid to appropriate problem identification in the first place?  Is there sufficient time and space for building of trusted relationships? |
|  | Resources, incentives and timeframe [3] | Incentives and motivation[3] | What are the motivations/incentives for participation in the CRE-IQI? Internal/external?  Are stakeholders given the opportunity to make a meaningful contribution to research?  Is the expected time-investment of stakeholders sufficiently clarified? |
|  |  | Changing conditions and flexibility[3] | Is the CRE-IQI sufficiently open and flexible to respond to changing conditions? (e.g. changing policy directives etc)  Have there been changes in the focus paid to addressing different CRE-IQI aims and objectives? |
|  |  | Resources and sustainability[3, 5] | Perceptions of the use of resources? What CRE-IQI resources do you use (reports, research publications, bi-annual meetings, newsletters, etc.)?  What is the sustainability of the innovation platform beyond the CRE-IQI, and of the partnerships? |
| Innovation platform:  - Outcomes[1]  - Performance[2] | Performance and outcomes | Performance and outcomes | How would you assess the CRE-IQI’s level of achievement in meeting its aims?  What do you see as the major outcome of the CRE-IQI?  To what extent have the principles enabled (or not) the CRE-IQI to achieve its aims/desired results?  What can we learn from the implementation of this principle for further work?  Other factors that supported or hindered these outcomes? Enablers or drivers of outcomes? |

*Footnotes: Not all interviewees will be asked all of the following questions, as the direction the interview takes will depend upon interviewees’ interests. This Interview Guide draws on a number of frameworks for evaluating innovation platforms[1-3], learning collaboratives[5] and principles-focused evaluation[4].*

1. Pali, P. and K. Swaans, *Guidelines for innovation platforms: Facilitation, monitoring and evaluation*. 2013, International Livestock Research Institute: Nairobi, Kenya.

2. Cadilhon, J.J., *A conceptual framework to evaluate the impact of innovation platforms on agrifood value chains development*, in *138th EAAE Seminar on Pro-poor Innovations in Food Supply Chains*. 2013: Ghent Belgium.

3. Boogaard, B.K., et al., *Critical Issues for Reflection When Designing and Implementing Research for Development in Innovation Platforms*. 2013, Wageningen University & Research Centre: The Netherlands.

4. Patton, M.Q., *Principles-focused evaluation: The GUIDE*. 2017: Guilford Publications.

5. Nix, M., et al., *Learning Collaboratives: Insights And A New Taxonomy From AHRQ’s Two Decades Of Experience.* Health Affairs, 2018. **37**(2): p. 205-212.
